# The effects of graded levels of calorie restriction: IX. Global metabolomic screen reveals modulation of carnitines, sphingolipids and bile acids in the liver of C57BL/6 mice

**DOI:** 10.1111/acel.12570

**Published:** 2017-01-31

**Authors:** Cara L. Green, Sharon E. Mitchell, Davina Derous, Yingchun Wang, Luonan Chen, Jing‐Dong J. Han, Daniel E. L. Promislow, David Lusseau, Alex Douglas, John R. Speakman

**Affiliations:** ^1^ Institute of Biological and Environmental SciencesUniversity of AberdeenAberdeenUK; ^2^ State Key Laboratory of Molecular Developmental BiologyInstitute of Genetics and Developmental BiologyChinese Academy of SciencesChaoyangBeijingChina; ^3^ Key Laboratory of Systems Biology, Innovation Center for Cell Signaling NetworkInstitute of Biochemistry and Cell BiologyShanghai Institute of Biological SciencesChinese Academy of SciencesShanghaiChina; ^4^ Key Laboratory of Computational BiologyChinese Academy of Sciences‐Max Planck Partner Institute for Computational BiologyShanghai Institutes for Biological SciencesChinese Academy of SciencesShanghaiChina; ^5^ Department of Pathology and Department of BiologyUniversity of WashingtonSeattleWAUSA

**Keywords:** aging, bile acid, calorie restriction, carnitine, Metabolomics, sphingolipid

## Abstract

Calorie restriction (CR) remains the most robust intervention to extend lifespan and improve health span. Using a global mass spectrometry‐based metabolomic approach, we identified 193 metabolites that were significantly differentially expressed (SDE) in the livers of C57BL/6 mice, fed graded levels of CR (10, 20, 30 and 40% CR) compared to mice fed *ad libitum* for 12 h a day. The differential expression of metabolites also varied with the different feeding groups. Pathway analysis revealed that graded CR had an impact on carnitine synthesis and the carnitine shuttle pathway, sphingosine‐1‐phosphate (S1P) signalling and methionine metabolism. S1P, sphingomyelin and L‐carnitine were negatively correlated with body mass, leptin, insulin‐like growth factor‐ 1 (IGF‐1) and major urinary proteins (MUPs). In addition, metabolites which showed a graded effect, such as ceramide, S1P, taurocholic acid and L‐carnitine, responded in the opposite direction to previously observed age‐related changes. We suggest that the modulation of this set of metabolites may improve liver processes involved in energy release from fatty acids. S1P also negatively correlated with catalase activity and body temperature, and positively correlated with food anticipatory activity. Injecting mice with S1P or an S1P receptor 1 agonist did not precipitate changes in body temperature, physical activity or food intake suggesting that these correlations were not causal relationships.

## Introduction

Human lifespan continues to increase at, on average, two years per decade (Kirkwood, 2008). This increase in lifespan has persisted over the last two centuries and has resulted in age becoming a major risk factor in the most prevalent clinical conditions, which include cancer, cardiovascular disease and dementia. To attenuate these age‐associated clinical conditions, many approaches, in a variety of model organisms, have been investigated (reviewed in Fontana *et al*., 2010). Although the effects of aging have been partially reversed though genetic mutations, drugs and environmental changes, calorie restriction (CR) is the only nongenetic technique that consistently increases lifespan (Tosato *et al*., 2007; Speakman & Mitchell, 2011). The impacts of CR have been well documented in rodents, where it improves health span and increases lifespan (Richard Weindruch, 1988; Speakman *et al*., 2016). The beneficial effects of CR are observed in many other species, ranging from nematode worms to nonhuman primates (Speakman & Mitchell, 2011; Colman *et al*., 2014).

The mechanisms that result in CR induced increases in lifespan and decreases in age‐associated diseases have yet to be fully explained. This is in part due to complexity of the aging process and the shift from healthy aging to pathological disease‐associated aging. In rodents, an increase in CR causes a corresponding increase in lifespan, a relationship which suggests a close link between aging and nutrient metabolism (Speakman *et al*., 2016).

The liver is a key organ in the regulation of lipid and glucose homeostasis. Relative to other organs, the liver does not display marked changes with age; however, it is associated with a loss of hepatic volume and hepatic perfusion (Schmucker & Sanchez, 2011). Liver cellular structure shows age‐associated increases in cell size and a decrease in the number of mitochondria, in both rodents (Herbener, 1976) and humans (de la Cruz *et al*., 1990). The destabilization of mitochondrial processes is thought to be an early marker, or possibly a cause, of aging and age‐related disorders (Rector *et al*., 2010). These age‐associated deteriorations have a part to play in the marked increase in mortality from liver disease in the elderly (Regev & Schiff, 2001).

Several important functions in the liver are regulated by insulin, including protein and lipid synthesis, lipid storage, glycolysis, glucose storage, gluconeogenesis and the inhibition of ketogenesis (Shaham *et al*., 2008). With age, insulin resistance and visceral adiposity increase, this can cause an increase in inflammation and interfere with systemic glucose and lipid metabolism (Shoelson *et al*., 2006; Sepe *et al*., 2011). The increase and redistribution of adipose tissue increase leptin and insulin resistance, which effects the regulation of energy balance (Friedman, 2011). This increase in adiposity is in part thought to be due to a shift towards lipogenesis. CR has been suggested to ameliorate age‐associated changes in the liver by decreasing lipogenesis, leptin and free fatty acids (Kuhla *et al*., 2014).

We would expect an increase in the level of CR to have an incremental effect on liver metabolism, as many liver processes are regulated through nutritional signals such as carbohydrate and amino acid levels (Meijer, 2003; Wei *et al*., 2007). We performed an untargeted metabolomic analysis on the livers of calorie restricted and control mice using liquid chromatography–mass spectrometry (LC‐MS). Using restriction levels of 10–40% allowed us to identify metabolites which show a graded response to the intensity of CR, as we expected these metabolites to be most significantly related to lifespan‐associated changes, due to the linear effect of the level of restriction on longevity (Speakman *et al*., 2016).

## Results

### CR induced changes in the liver metabolome

**Figure 1:**
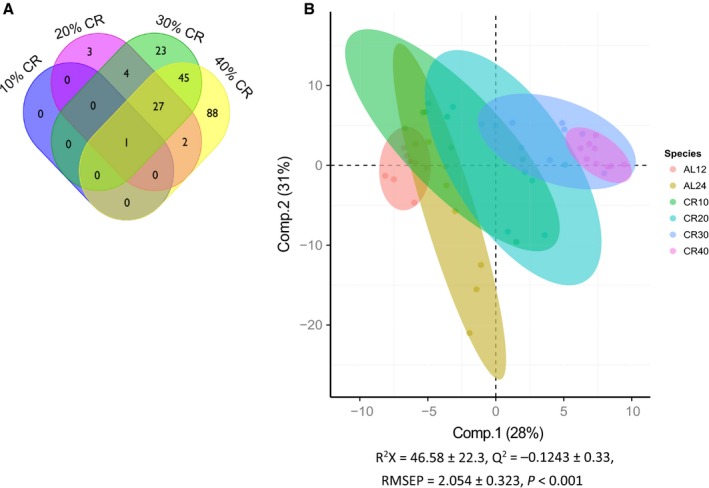
(A) Venn diagram showing the overlap between significantly differentially expressed metabolites. Each CR group (10, 20, 30, and 40) was compared to the 12AL, adjusted *P* ≤ 0.05. (B) Orthogonal partial least‐squares discriminate analysis (O‐PLS‐DA) demonstrates the differentiation effect of diet in metabolomic profiles. The O‐PLS‐DA plot showed significant separation among samples on the basis of the model quality parameters: R^2^X, Q^2^, RMSEP. The first two principal components explained 59% of the data. Adjusted *P* ≤ 0.05.

Male mice were aged 20 weeks when they began CR for 12 weeks. Two control groups, 12‐ and 24‐h *ad libitum* access to food (12AL and 24AL, respectively) were used, plus four levels of CR; 10, 20, 30 and 40% restriction from baseline food intake. 12AL was used as the control group for all comparisons, food was given at 1830 h (as with the CR mice) and removed 12 h later to alleviate the ‘time since last meal’ effect, as all mice had been food deprived for at least 7.5 h before culling. We found 886 unique metabolites, of which 193 were significantly differentially expressed (SDE) between the four CR levels relative to 12AL (adjusted *P*‐value ≤0.05). As the level of restriction increased, the number of SDE metabolites also rose (Table 1). Of the 193 metabolites, 88 were unique to 40CR, 23 were unique to 30CR, and three were unique to 20CR (Fig. 1A). Of the SDE 20CR metabolites, 92% overlapped with 30 and 40CR, and 73% of the SDE 30CR metabolites were also found in 40CR (All SDE metabolites are detailed in Table S1). We found one metabolite that was SDE by all levels of CR, putatively identified as D‐ribonate, a by‐product of glycogen breakdown. We performed an orthogonal signal correction partial least‐squares discriminant analysis (O‐PLS‐DA) on metabolites that were SDE between 12AL and at least one CR group using Fisher's exact test (Fig. 1B, adjusted *P*‐value ≤0.05). The model indicated that around 60% of the variance in the metabolites could be explained by the dietary treatment group (single‐sample t‐test between model parameters and permuted parameters, *P*‐value <0.001).

**Table 1:** Number of significantly differentially expressed (SDE) metabolites, relative to 12AL control for each CR treatment level and the 24AL group. BH adjusted corrected for false discovery rate *P* ≤ 0.05

	24AL	10CR	20CR	30CR	40CR
Up	0	0	21	58	88
Total	0	1	37	100	163
Down	0	1	16	42	75

### Metabolic pathway changes in response to CR

**Figure 2:**
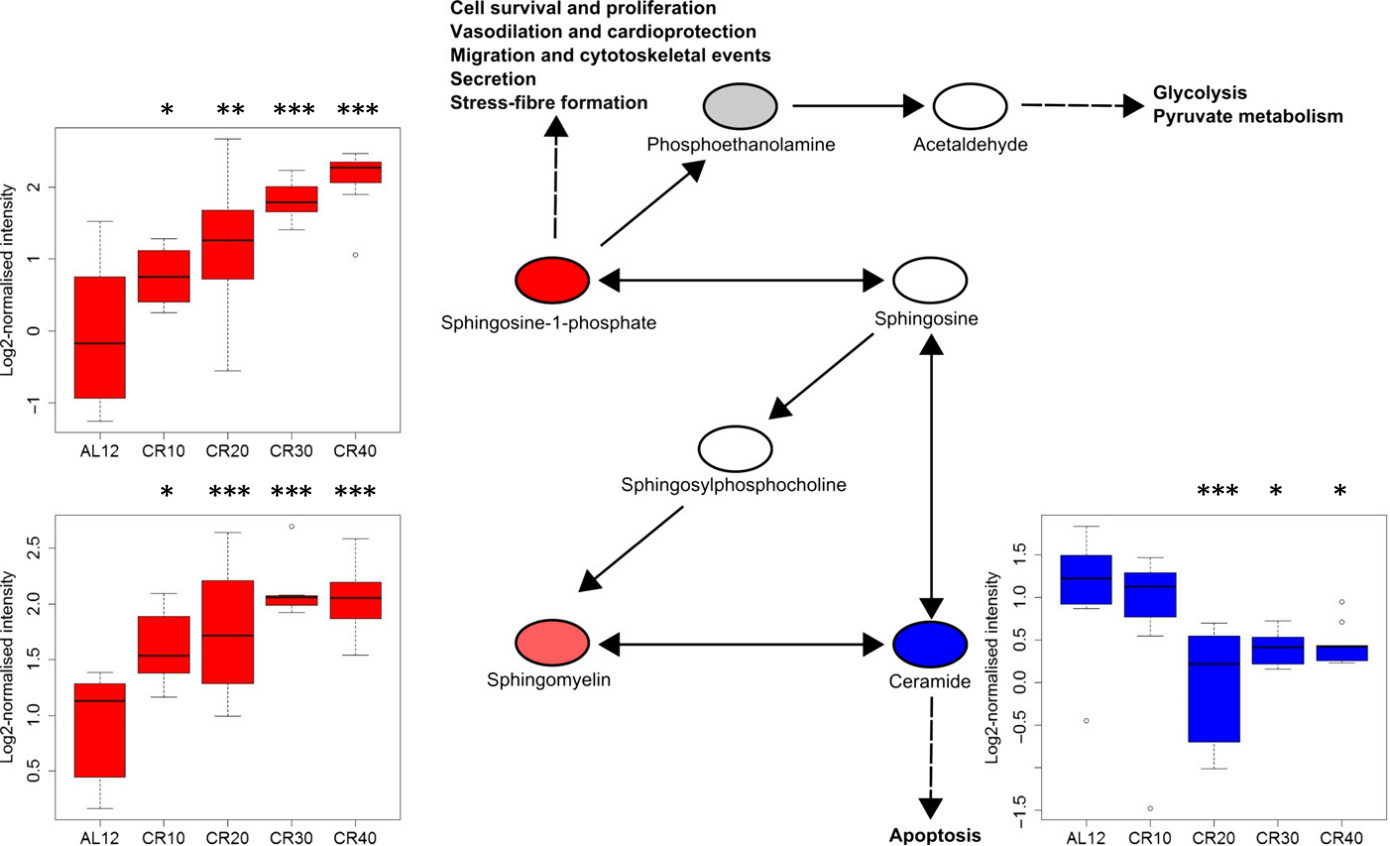
Sphingosine‐1‐phosphate and precursors pathway indicating cellular effects. Boxplots indicate intensity level of each metabolite across groups (*n *= 7–9), showing outliers, min, max and interquartile range. Red = upregulated, blue = downregulated, grey = not significantly changed, white = undetected in sample. *P*‐values are from SDE metabolite analysis of each CR group relative to 12AL. * *P* < 0.05, ** *P* < 0.01, *** *P* < 0.001.

**Figure 3:**
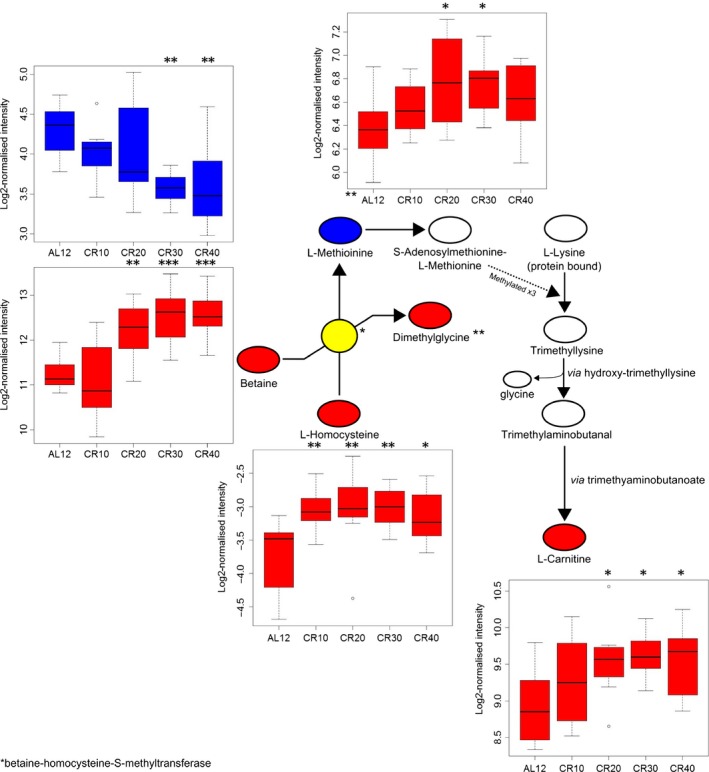
Pathway showing the production of L‐carnitine from L‐methionine and L‐lysine. Boxplots indicate intensity level of each metabolite. Red = upregulated, blue = downregulated, grey = not significantly changed, white = undetected in sample. *P*‐values are from SDE metabolite analysis of each CR group relative to 12AL. * *P* < 0.05, ** *P* < 0.01, *** *P* < 0.001.

We identified metabolic pathways using ingenuity pathway analysis (IPA) and *mummichog*. We inputted identified SDE metabolites into IPA which gave us significantly altered metabolic pathways (Table 2) and metabolites (Table S2), relative to 12AL. Ceramide and sphingosine‐1‐phosphate (S1P) signalling featured in the top three pathways for all four levels of CR (Fig. 2, *P *< 0.001 for all treatment levels). In 20, 30 and 40CR, the methionine salvage II pathway was in the top three pathways (*P* < 0.001 for all groups). At 30 and 40CR, L‐carnitine biosynthesis (Fig. 3) was significantly altered (*P* = 0.001 and *P* = 0.003, respectively) and all the identified metabolites (*n *= 3) were upregulated. In the top ten increased metabolites for 40CR relative to 12AL, three metabolites were involved in carnitine metabolism/signalling; L‐palmitoylcarnitine (*P* ≤ 0.001), acetyl‐L‐carnitine (*P* = 0.003) and propionylcarnitine (*P* < 0.001). At 40CR, sphingomyelin metabolism was significantly altered relative to 12AL (*P* < 0.001), with 50% of metabolites upregulated and 17% downregulated.

**Table 2:** For each CR group in comparison with the 12AL group. The percentage of metabolites up‐ and downregulated for each of the top six canonical pathways calculated using IPA. *P*‐values are based on the number of metabolites found in liver samples relative to total number of molecules in known metabolic pathway

Pathway	40 CR			30 CR			20 CR			10 CR		
	Up	Down	*P*‐value	Up	Down	*P*‐value	Up	Down	*P*‐value	Up	Down	*P*‐value
Ceramide signalling	50%	25%	<0.001	50%	25%	<0.001	50%	25%	<0.001	50%	25%	0.001
S‐1‐P signalling	50%	25%	<0.001	50%	25%	<0.001	50%	25%	<0.001	50%	25%	0.001
Methionine salvage II	33%	17%	<0.001	50%	17%	<0.001	50%	17%	<0.001			
Sphingomyelin metabolism	50%	17%	<0.001									
L‐carnitine biosynthesis	50%	0%	0.003	50%	0%	<0.001						
Phospholipases	0%	50%	0.003									
Glycine betaine degradation				23%	23%	0.001	23%	23%	0.002			
Cysteine biosynthesis III				23%	23%	0.001						
Sphingosine and S‐1‐P metabolism							40%	10%	0.001			
NGF signalling							17%	17%	0.006			
Phenylalanine degradation IV										28%	17%	0.001
Glutamine biosynthesis I										67%	0%	0.002
Asparagine biosynthesis I										43%	14%	0.003
Methionine degradation I										38%	13%	0.004

We used metabolite set enrichment to obtain putative metabolite identifications and significantly changed pathways based on mass–charge (*m/z*) ratios using *mummichog* (Table 3). Metabolites for each comparison (CR relative to 12AL) were considered significant if *P* ≤ 0.05 and were evaluated alongside a reference list of all metabolites found in the sample. In 40CR, the carnitine shuttle pathway, several amino acid pathways, vitamin B3 metabolism and fatty acid β‐oxidation were the most significantly altered pathways relative to 12AL. For 30CR, the carnitine shuttle pathway was also significant relative to 12AL, in addition to pyrimidine, amino acid and butanoate metabolism. Carnitine shuttling was the most significantly altered pathway at 20CR, which also included fatty acid β‐oxidation. Saturated fatty acid β‐oxidation was significant for both 20 and 40CR (*P* = 0.047 and *P* = 0.014 respectively) which is also linked to the shuttle of long‐chain fatty acids across the mitochondrial membranes by carnitines. The pathways significant for 10CR included amino acid and glycerophospholipid metabolism (*P* = 0.02).

**Table 3:** Significant pathways for each CR level relative to 12AL from the pathway enrichment analysis computed by *mummichog*. Based on the m/z scores of significant metabolites from the empirical Bayes linear modelling from the 40CR – 12 AL comparison (*P* ≤ 0.05)

Pathways	Overlap size	Pathway size	*P*‐value
40CR			
Carnitine shuttle	8	11	0.00226
Lysine metabolism	11	19	0.00337
Methionine and cysteine metabolism	12	26	0.01553
Drug metabolism – other enzymes	3	4	0.0231
Vitamin B3 (nicotinate and nicotinamide) metabolism	6	12	0.02696
Porphyrin metabolism	4	7	0.03105
Saturated fatty acids beta‐oxidation	2	2	0.04654
Glycine, serine, alanine and threonine metabolism	13	32	0.04987
30CR			
Carnitine shuttle	7	11	0.00522
Pyrimidine metabolism	11	23	0.00891
Lysine metabolism	9	19	0.01347
Glutamate metabolism	6	11	0.01369
Butanoate metabolism	6	11	0.01369
20CR			
Carnitine shuttle	6	11	0.0016
Drug metabolism – cytochrome P450	9	27	0.00733
Glycerophospholipid metabolism	7	20	0.00889
Saturated fatty acids beta‐oxidation	2	2	0.01387
Glycosphingolipid metabolism	6	18	0.01742
Vitamin E metabolism	4	11	0.02752
Selenoamino acid metabolism	3	7	0.02998
10CR			
Lysine metabolism	6	19	0.01463
Glycerophospholipid metabolism	5	20	0.05926
Drug metabolism – other enzymes	2	4	0.06117
Pyrimidine metabolism	5	23	0.11322
Tryptophan metabolism	8	40	0.12019

### Twelve‐hour *ad libitum* vs. 24‐h ad libitum

No metabolites were SDE between 24AL and 12AL (adjusted *P* ≤ 0.05). When we relaxed the significance criteria and used the unadjusted *P*‐value, we found 41 SDE metabolites that were increased and 36 that were decreased relative to 12AL (*P* ≤ 0.05). Using IPA, one metabolic pathway, glutamine degradation I, was found to be altered in 24AL relative to 12AL (*P* = 0.018).

### Correlations between CR‐dependent metabolites and CR‐dependent physiological traits

**Figure 4:**
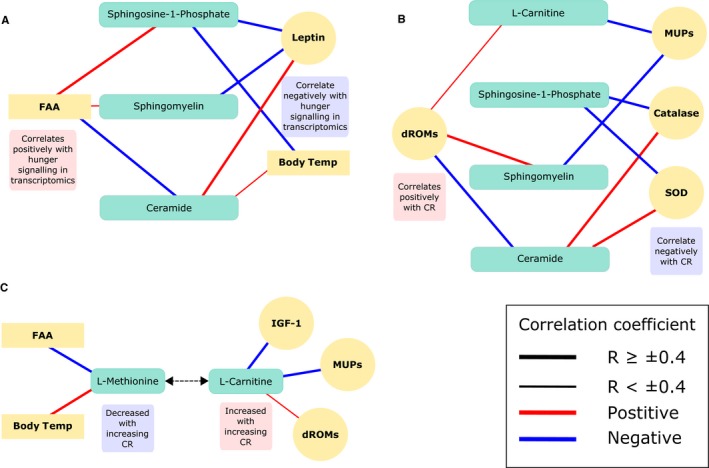
Overview of SDE metabolites (BH adjusted *P* < 0.05) which exhibited a graded response to CR and correlations with physiological parameters (Pearson's correlations adjusted *P* ≤ 0.05). (A) Sphingolipids and their relationship with parameters involved in hunger signalling. (B) Sphingolipids and L‐carnitine and the relationship with markers of oxidative stress and antioxidants. (C) L‐carnitine and its precursor L‐methionine and the relationship with physiological parameters, hormones and oxidative stress markers. FAA = food anticipatory activity, MUPs = major urinary proteins, SOD = superoxide dismutase, dROMs = derivatives of reactive oxygen metabolites. Connections between metabolites and other measures indicate correlations, where red equals a positive and blue a negative correlation.

To explore the metabolites that had a graded response further, we correlated them (Pearson's correlation) with circulating hormone levels (Table S3), major urinary proteins (MUPs) and markers of oxidative stress (Table S4), measures of which are detailed in our previous paper (Mitchell *et al*., 2015b). We found that circulating levels of leptin correlated negatively with S1P and sphingomyelin (SM) and positively with ceramide (Fig. 4A, S1P *r *= −0.431, *P* = 0.043 and SM *r *= −0.401, *P* = 0.048, Cer *P* = 0.597, *P* = 0.002). These sphingolipids, in addition to the facilitator of fatty acid oxidation, L‐carnitine, correlated negatively with IGF‐1 (S1P *r *= −0.597, *P* = 0.001, SM *r *= −0.595, *P* = 0.001 and L‐c ‐0.429, *P* = 0.027) and MUPs, which are a marker of male reproductive investment (Fig. 4B, S1P *r *= −0.646, *P* < 0.001, SM *r *= −0.557, *P* = 0.003 and L‐c *r *= −0.510, *P* = 0.004). We also found that L‐carnitine and several other carnitine metabolites correlated positively with dROMs (derivatives of reactive oxygen metabolites), which is a measure of oxidative stress‐related metabolites in the blood (Fig. 4C, Table S5). Leptin levels were significantly associated with levels of white adipose tissue. Residual leptin (adjusted for white adipose tissue level) was not significantly associated with S1P, sphingomyelin or ceramide levels (S1P *r *= 0.07, *P* = 0.66, SM *r *= −0.11, *P* = 0.50 and Cer *r *= 0.19, *P* = 0.25).

Additionally, we correlated these metabolites with physiological data (Mitchell *et al*., 2015a,c, 2016a). S1P and sphingomyelin were negatively correlated with body mass (S1P *r *= −0.761, *P* < 0.001 and SM *r *= −0.453, *P* = 0.019) and positively correlated with food anticipatory activity (activity in the 2 h before food supply: FAA) (S1P *r *= 0.771, *P* < 0.001 and SM *r *= 0.365, *P* = 0.024), whereas ceramide correlated in the opposite direction (BM *r *= 0.550, *P* = 0.003 and FAA *r *= −0.412, *P* = 0.015). Levels of S1P strongly negatively correlated with Body temperature (Tb) (*r *= −0.770, *P* < 0.001), previously we found that Tb over the last 20 days of CR correlated negatively with the level of restriction.

The conjugated bile acids taurocholic acid (30CR, *P* = 0.047 and 40CR, *P* = 0.001) and taurochenodeoxycholate (*P* = 0.028) were SDE at higher levels of restriction and showed a graded response to CR. However, they did not correlate with any of the hormones or oxidative stress parameters.

### Injection of S1P and S1PR1 agonist does not cause a change in Tb or food intake

Despite the relationship between CR and S1P, and S1P with body mass, FAA and Tb, we found no indication that peripheral injection of S1P, or an S1P receptor agonist (SEW2871), had any effect on Tb or food intake. Single 100 ng and 200 ng (injected as 100 μL and 200 μL, respectively) injections were given in triplicate on different days at 1000 h (*n *= 5). Model selection indicated that using activity alone gave the best model when using Tb as the dependent variable (Table S6) and using the intercept alone gave the best model for food intake (Table S7). Treatment as a fixed effect did not significantly explain Tb (*P* = 0.558, Table S8 and Table S9), nor did using treatment as a random effect with ID (Table S6, model 1). Treatment or activity as fixed effects did not significantly affect food intake, in either the light or dark period, nor did the interaction between treatment and activity (Table S8 and S9).

## Discussion

### Graded CR provides novel insights into the effects of CR

In contrast with earlier studies, which typically included a single treatment level, we used graded CR treatment groups (but see also Nogueira *et al*., 2012; Kim *et al*., 2016). This design enabled us to show not only that there was an increasing number of SDE metabolites with increasing CR, but also that many of these metabolites, including L‐carnitine, S1P and taurocholic acid, were increasingly upregulated with increasing CR.

### The increase in SDE metabolites was independent of liver structure

We have previously shown that liver mass decreases by 17.5, 27.6, 25.6 and 27.7% at restriction levels of 10, 20, 30 and 40%, respectively, relative to the 12AL control (Mitchell *et al*., 2015c). These losses were significant between 12AL and the CR groups, but not between CR groups. In contrast, we observed a rise in the number of SDE metabolites relative to 12 AL as restriction increased. Given that the number of SDE metabolites continued to rise with an increase in CR, and that liver mass was not responsive to the level of restriction, the changes in metabolic pathways appeared independent of tissue mass loss. This supports the idea that although liver morphology is relatively protected with CR and age, liver function is plastic when confronted with such challenges.

### Lipid and glycolysis pathway remodelling occurs in the liver metabolome with CR

Previous metabolomic analyses on the livers of C57BL/6 CR mice suggest that glycolysis, fatty acid oxidation and amino acid pathways are significantly altered (Collino *et al*., 2013; Fok *et al*., 2014; Jové *et al*., 2014). These changes were consistent with studies that indicate metabolic reprogramming through the shift from lipogenesis to lipolysis during CR is beneficial in both male and female C57BL/6 mice (Bruss *et al*., 2010; Kuhla *et al*., 2014). In our study, we found that several pathways involved in fatty acid metabolism were altered with CR, which further supports these hypotheses. In terms of a graded response, which is important when we consider the most influential pathways on lifespan, short‐term CR appeared to have most effect on sphingolipid and carnitine metabolism. However, methionine, cysteine, phenylalanine, glutamine, asparagine, lysine and tryptophan pathways appeared modulated at varying levels of CR in the present analysis. This is in concordance with studies that found changes in a wide range of different amino acids in the liver with CR in both C57BL/6 and DBA2 mice (Collino *et al*., 2013; Mitchell *et al*., 2016b). Pathway analyses did not reveal that amino acid pathways were particularly altered with CR, this is in contrast with the results of (Mitchell *et al*., 2016b); however, in that study CR was lifelong, and the most significant amino acid changes in C57BL/6 mice appeared in the females. As our mice were male and the duration of restriction shorter, it may be that less significant amino acid changes are seen after 3 months of restriction. We identified a decrease in D‐ribonate as the only consistently SDE metabolite at all CR treatment levels relative to 12AL. This indicated that glycogen breakdown in the livers of CR mice was reduced after 3 months of CR, perhaps because glycogen stores were depleted during the initial phase of restriction. This would also be consistent with a shift towards lipolysis, which supports the idea that during CR, there is an increase in oxidation of fatty acids as opposed to glucose utilization. This compliments transcriptomic liver data from previous studies, which indicate CR increases fatty acid mobilization and metabolism in several strains of mice and rats (Selman *et al*., 2006; Plank *et al*., 2012; Collino *et al*., 2013). Three key metabolic groups showed increased responses to the levels of restriction. Independently, these have all been associated previously with CR and aging.

### S1P and ceramide

S1P and ceramide are associated with the beneficial effects of CR in male Wistar rats and C57BL/6 mice (Collino *et al*., 2013; Babenko & Shakhova, 2014) and lifespan regulation in mammals (Lightle *et al*., 2000; Huang *et al*., 2014). They are thought to have evolved to protect cells from environmental stress (Van Brocklyn & Williams, 2012). Reactive oxygen species (ROS) can act as a switch for the interconversion of these metabolites, and during excess oxidative stress, ceramide is produced and sphingosine kinase 1 is degraded, reducing levels of S1P and tipping the balance towards apoptosis (Van Brocklyn & Williams, 2012). S1P and sphingomyelin (increased with CR) correlated negatively with circulating leptin levels, whereas their precursor, ceramide (decreased with CR), was positively correlated with leptin (Fig. 4A). Leptin was reduced with increasing levels of restriction and was strongly positively correlated with adiposity (Mitchell *et al*., 2015b). As leptin is a key regulator of long‐term energy balance, its reduction during CR may be antagonistically regulating S1P and ceramide (Rosen & Spiegelman, 2006). Although no direct relationship between leptin signalling and S1P or ceramide has yet been discovered, and we found the relationship between these metabolites and leptin may be completely explained by adiposity, low levels of ceramide have been associated previously with reduced mass and improved insulin signalling in the liver and muscle tissue in *ob/ob* and obese mice (Yang *et al*., 2009). Moreover, S1P‐stimulated cells have shown a reduction in insulin‐induced leptin synthesis in rats (Jun *et al*., 2006). Our study suggests that this relationship may be important in the beneficial effects or CR, particularly as the pathway shows a graded response to CR levels.

It is possible that alongside leptin and insulin pathways, S1P and ceramide may have an important role in signalling the energy status of the animal in the brain through specific kinases and phosphatases (van Echten‐Deckert *et al*., 2014). Previous work suggested that in the hypothalamus, S1P regulates energy homeostasis in rats. Microinjection of S1P into the third ventricle of rats increased Tb and energy expenditure, and microinjection of S1PR1 agonist SEW2871 decreased food intake and activated leptin signalling (Silva *et al*., 2014). We also observed correlations between these metabolites and aspects of physiology of the CR mice, including Tb and FAA, but the correlation with respect to Tb was opposite that previously observed in the brain. One distinctive response in our mice was a large increase in FAA, and in addition the level of FAA was correlated with transcriptomic levels of several key hunger signalling neuropeptides in the hypothalamus (Derous *et al*., 2016). The association of S1P and Tb is potentially significant in the context of the potential causal relationship between Tb and longevity. In the longest‐lived rodent, the naked mole‐rat (*Heterocephalus glaber*), low Tb is thought to contribute to its longevity. Furthermore long‐lived Ames dwarf mice have a lower Tb than wild‐type mice, potentially related to deficiencies in thyroid‐stimulating and growth hormones and lower insulin levels (Keil *et al*., 2015). Hcrt‐UCP2 mice, which overexpress UCP2, also have reduced Tb and increased lifespan, thought to be related to increased energy efficiency (Conti *et al*., 2006). We also saw that Tb correlated with body fat, leptin and IGF‐1. Due to its relationship with Tb and its role as a bioactive signalling molecule, we hypothesized that S1P may be a peripheral signal involved in the stimulation of these hunger signalling pathways alongside leptin, insulin and IGF‐1. To explore whether these correlations reflected an underlying causal role for S1P signalling, we performed an experiment to evaluate the role of S1P on these traits. However, we found that peripherally injecting S1P (intraperitoneally) had no effect on Tb, activity level or food intake. The lack of effect seen may indicate that S1P is not causally linked to the responses observed under CR, such as decreased physical activity, increased FAA and lowered Tb. However, this could be due to the peripheral injection of S1P not reaching a certain threshold in the liver to be effective, compared to the previous study where S1P was injected directly into the brain of rats (Silva *et al*., 2014). The physical changes we see with CR, along with the increase in S1P, may be downstream of other CR related responses such as lowered leptin levels. The observed increase in S1P may be liver‐specific and acting to promote cell survival, immune cell trafficking, cell motility and histone modification (van Echten‐Deckert *et al*., 2014; Gomez‐Muñoz *et al*., 2015). The increase in S1P we see with CR may also be acting as a switch to reduce ceramide, which can promote inflammation through NF‐ƙB (nuclear factor kappa‐light‐chain enhancer of activated B cells) in liver mitochondria, and conversion into S1P may act to attenuate this response (Gomez‐Muñoz *et al*., 2015).

It appears that a reduction in the ceramide: S1P ratio may be beneficial as ceramide is associated with rapid ROS production to initiate apoptotic cell death through TNF‐α signalling (Lecour *et al*., 2006; Dumitru *et al*., 2007). Sphingosine kinase 1, which converts sphingosine to S1P, is thought to act as a switch enzyme that can affect the S1P/ceramide ratio downstream of ROS in stress signalling (Van Brocklyn & Williams, 2012). However, we found that ceramide was positively correlated with superoxide dismutase, a major antioxidant defence system against superoxide anions which cause cell stress (Fukai & Ushio‐Fukai, 2011) and negatively correlated with reactive oxygen metabolites (ROMS) in the blood. It is unclear whether bioactive ceramide had a direct effect on the level of ROMS in the blood, as measures of oxidative stress are tissue‐dependent (Xu *et al*., 2014).

As with ceramide we found that MUPs significantly decreased with increasing CR, MUPs are nonvolatile components found in urine, which are a marker of reproductive investment (Mitchell *et al*., 2015b). This result is likely to be unique to male mice, as they produce significantly more MUPs in their urine than female mice, which are stimulated by testosterone, growth hormone and thyroxine (Knopf *et al*., 1983). MUPs were also negatively correlated with S1P, sphingomyelin and L‐carnitine. MUPs are thought to cause oxidative stress during their synthesis in male mice. These results suggest that in addition to metabolic remodelling, these metabolites and their associated pathways may be augmenting reductions in reproductive investment to conserve energy and decrease ROS production.

### Circulating hormone levels may cause an increase in L‐carnitine and decrease in L‐methionine

Pathway analysis indicated that fatty acid metabolism (increase in β‐oxidation), fatty acid transport (increase in the carnitine shuttle) and fatty acid messengers (S1P and ceramide) were significantly altered, even at lower levels of CR. We also found that all identified carnitines and carnitine derivatives were increased with CR, and this has implications for longevity, as in *Drosophila* L‐carnitine shows a highly significant decline with age (Hoffman *et al*., 2014). L‐carnitine shuttles fatty acids across the mitochondrial membrane, where they can be processed by β‐oxidation enzymes to ultimately generate ATP (Flanagan *et al*., 2010; Pekala *et al*., 2011). Studies have shown that L‐carnitine supplementation has positive effects in obese and diabetic humans, as well as benefits in cats and mice (Levin *et al*., 1999; Blanchard *et al*., 2002; Mingorance *et al*., 2012). The L‐carnitine biosynthesis pathway was significantly upregulated at 30 and 40CR, and all metabolites detected in this pathway showed a graded response (Fig. 3). It is thought that a high proportion of L‐carnitine is endogenously produced through L‐lysine and L‐methionine (Krajcovicová‐Kudlácková *et al*., 2000). Although the metabolite precursors of methionine production (L‐homocysteine and betaine) increased with restriction, levels of methionine were decreased. One possibility is that methionine was decreased due to incorporation into the L‐carnitine biosynthesis pathway, thereby improving fatty acid transport efficiency across the mitochondrial membrane.

In the skeletal muscle of rats, carnitine supplementation increases IGF‐1 plasma levels, which activates the IGF‐1/PI3K/Akt signalling pathway (Keller *et al*., 2013). However, we saw that L‐carnitine correlated negatively with levels of IGF‐1 as well as MUPs, although we measured L‐carnitine in the liver. A study using mouse embryonic fibroblasts showed that although low levels of L‐carnitine activated the IGF‐1/PI3K/Akt pathways, high levels of L‐carnitine decreased the expression of *IGF‐1* and significantly inhibited the pathway (Ge *et al*., 2015). This may indicate that carnitine regulation of IGF‐1/PI3K/Akt pathways is dose dependent. Although we did not measure the direct effect of L‐carnitine on the IGF‐1/PI3K/Akt pathway, we did see that with increasing CR, IGF‐1 decreased and L‐carnitine increased. The regulation of the IGF‐1/PI3K/Akt pathways is pertinent to nutritional lifespan studies as direct and indirect targets of Akt include mTOR and FOXO. Inhibition of mTOR increases lifespan in several species and can be reduced through CR (Navé *et al*., 1999; Johnson *et al*., 2013).

### Increasing bile acids may be linked to the control of metabolic pathways

As CR puts greater demands on the metabolism of lipids, we expected metabolites involved in fatty acid metabolism to be increased. This not only held true for the carnitine family, but also for bile acids. Bile acids break down large fat globules into smaller droplets and promote their absorption through formation of micelles. This helps stimulate energy metabolism and improves insulin sensitivity (Chiang, 2013). When cholesterol builds up in the liver, it can cause damage through fatty deposits, which cause inflammation (Zheng *et al*., 2008), but this process can be alleviated through the production of bile acids for which cholesterol is a precursor (Javitt, 1994). Bile acids also act as signalling molecules, which can regulate lipid and glucose metabolism through G protein‐coupled receptors in the liver (Qi *et al*., 2015). The bile acids taurocholic acid and taurochenodeoxycholate showed a significant graded increase with rising CR. Increasing age is associated with reduced bile acid synthesis in rodents (Ferland *et al*., 1989) and in humans and is thought to be related to the growth hormone/IGF axis (Bertolotti *et al*., 2007). CR has been shown to reverse the decline in the bile acid synthesis pathway with age in female C57BL/6 mice (De Guzman *et al*., 2013). Bile acids are also ligands for the farnesoid X receptor (FXR). FXR is important in controlling several metabolic pathways including lipid and glucose homeostasis and modulating liver regeneration, suggesting that FXR‐induced changes through bile acid activation may underpin some beneficial effects of CR (Zhang & Edwards, 2008). In conjunction with the increase in the carnitine shuttle pathway and possible nutritional modulation through the bioactive signalling molecule S1P, bile acids may be crucial to metabolic remodelling during CR.

### Twelve‐hour ad libitum vs. 24‐h ad libitum

Underlying our analysis was an initial decision to use 12AL as our control, as opposed to the more traditionally used 24AL fed control. As the 24AL group may have eaten immediately prior to culling, the 12AL group allowed us to separate long‐term restriction from short‐term starvation and evaluate the ‘time since last meal’ effect. We demonstrated that the 12AL and 24AL feeding groups showed significantly different metabolomic profiles with 9% of metabolites significantly different (using unadjusted *P*‐values).

## Conclusion

In this study, we found that as the level of CR increased, the number of SDE metabolites also increased. It is important to note that we saw significant changes in many age‐associated metabolites, in relatively young mice after only three months of CR. This indicates that short‐term CR as opposed to lifetime CR is sufficient to initiate beneficial remodelling of liver metabolism in male mice. Pathway analysis showed that CR had the greatest effect on the carnitine shuttle and synthesis pathways and on phospholipid metabolism pathways. Interestingly, we did not see significant changes in amino acid pathways, although large changes in amino acids were seen in female mice in Mitchell *et al*., 2016b. As our mice were male, it may be that metabolic remodelling shows gender‐specific fuel preferences. In addition to the increase in bile acids, these results are consistent with the notion that during CR, the liver becomes much more efficient at breaking down lipids and shuttling long‐chain fatty acids to the mitochondria. Furthermore, all of these effects acted in a graded way to CR, highlighting their potential relevance to longevity. The pathways and metabolites we highlight may be modulating improvements in metabolism through promoting utilization of lipids during CR and regulating energy homeostasis. Correlative data indicate the S1P/ceramide axis may be working downstream of hunger signalling pathways such as leptin and insulin/IGF‐1; however, we demonstrated that peripherally it is unlikely to directly affect Tb, FAA or food intake.

## Methods

### Experimental design

Our data set consisted of metabolomic data from technical triplicates of 48 individual liver samples across six different feeding groups, including four CR groups and two AL‐fed groups. Male mice were allocated to six treatment groups: 12AL, 24AL and 10, 20, 30 and 40% CR. Sample size was eight for all groups except 30CR where *n *= 7 and 40CR where *n *= 9. The 12AL group was used as the control. We initiated CR at 20 weeks; this was performed to avoid any effect of CR on development while retaining effectiveness of increasing lifespan (Yu *et al*., 1985). Mice were fed CR (or AL) diets for 12 weeks, before being sacrificed at 32 weeks of age.

### Animals

Mice were purchased from Charles River (Ormiston, UK). All procedures were reviewed and approved by University of Aberdeen ethical approval committee and carried out under a Home Office issued licence compliant with the Animals (Scientific Procedures) Act 1986. This strain is already known to live longer under CR (Turturro *et al*., 1999; Swindell, 2012). More information on procedures and measures can be found in Data S10 and the first paper of this series (Mitchell *et al*., 2015c).

### Liver metabolite extraction

Individual frozen mouse liver samples (≈ 25 mg) were homogenized using an ULTRA‐TURRAX^®^ dispenser T‐25 Basic (IKA^®^, Staufen, Germany) at level 5 in 1000 μL of chloroform: methanol: water (1:3:1) at 4°C. Samples were agitated for 1 h at 4°C and centrifuged at 13 000 g for 3 min at 4°C. Supernatant was aliquoted into 180 μL samples and stored under argon at −80°C. Samples were analysed with LC‐MS using an Orbitrap™ Exactive™ mass spectrometer at the Glasgow Polyomics facility. The ZIC^®^‐pHILIC (zwitterionic ion chromatography–hydrophilic interaction chromatography) platform detects mainly polar molecules, and nonpolar lipids can be identified in the wash‐through. A panel of known common metabolites are run alongside samples. Raw peak intensities were obtained for each metabolite, and where possible metabolites were identified by their retention times, mass to charge ratio (m/z ratio) and comparison to standards. As part of quality control, blanks containing chloroform: methanol: water and a pooled sample containing all 48 samples were analysed alongside the liver samples.

### Data preprocessing

A total of 6550 features across all samples including adducts, fragments and metabolites were detected. We filtered these features to unique metabolite chromatographic peaks, which included 505 identified and 381 unidentified metabolites. Among 886 metabolite features, 1.9% of the metabolite intensities were unclassified due to either low metabolite concentration, poor ionization, machinery limitations including detection sensitivity and ion suppression or true lack of presence (Huan & Li, 2015). To fill in these missing values, we imputed using a random forest regression approach (Stekhoven & Bühlmann, 2012; Gromski *et al*., 2014) using the missForest package (Stekhoven, 2011) for metabolites which had peaks that were significant in at least one group. Raw peak intensities for each sample were normalized by centring around the median for each metabolite in each sample using the Metabolomics package (De Livera & Bowne, 2013), then log_2_‐transformed.

### Statistical modelling of differential metabolite expression

To detect SDE metabolites between treatment groups, an empirical Bayes moderated linear model was fitted to each metabolite (Smyth, 2004). The empirical Bayes approach shrinks the estimated sample variances by borrowing information from across metabolites. Comparisons across metabolite fold‐changes were made between each level of CR (10, 20, 30 and 40%) and 24AL relative to 12AL. *P*‐values for each comparison were adjusted using the Benjamini–Hochberg (BH) procedure using a false discovery rate of 5% (Benjamini & Hochberg, 1995; Ferreira, 2007). We used the package Devium to produce the O‐PLS‐DA plot and to complete the validation steps (Wanichthanarak *et al*., 2015). O‐PLS‐DA allows us to discriminate between groups in multivariate data (Westerhuis *et al*., 2010).To validate the O‐PLS‐DA, we carried out 1000 simulations on randomly permuted groups to compare the modelled data to random expectation. We generated a pseudo training set by splitting the data into a testing and training set.

### Biological pathway analysis

We assigned KEGG (Kyoto Encyclopaedia of Genes and Genomes), HMDB (Human Metabolome Database), CAS (Chemical Abstracts Service) and CID (PubChem Compound Identifier IDs) IDs to 432 of the 505 identified metabolites. To infer biological meaning from our identified SDE metabolite set, we used IPA. This allowed us to determine whether the pathways were significantly altered relative to the 12AL control, based on the number of metabolites in each pathway and their abundances. There were 147 metabolites that were SDE between the CR groups and 12AL (unadjusted *P* ≤ 0.05) and had the IDs mentioned above. IPA takes advantage of the Ingenuity^®^ Knowledge Base, a repository of biological and chemical information manually curated from published literature, public and private databases and in‐house expert knowledge.

We used *mummichog* to analyse biological activity in each treatment group from the raw m/z ratios (Li *et al*., 2013). *Mummichog* looks up m/z features in its human network model, an integrated model from KEGG, UCSD BiGG and Edinburgh Model (Li *et al*., 2010). *P*‐values are based on the number of significant metabolite hits per pathway, and identifications are made based on the metabolites in those enriched networks. The inherent differences and biases that exist within the IPA and mummichog databases expectedly lead to slightly different pathway results.

### Correlations with physiological parameters

Circulating hormone levels and measures of oxidative stress were correlated (Pearson's correlation) with all metabolite intensities for each individual mouse. The average FAA of the last 20 days of treatment, core Tb over the final two weeks and the final body mass were also correlated. Associated *P*‐values for each correlation were adjusted using the BH procedure using a false discovery rate of 5%.

#### S1P experiment

Information on experimental set‐up can be found in Data S11.

Single intraperitoneal S1P (100 ng and 200 ng), SEW2871 (100 ng and 200 ng) or saline control injections (triplicates) were performed at 16 weeks. Injections were performed at 1000 h, after weighing. S1P and SEW2871 were obtained from the Cayman Chemical Company (Ann Arbor, MI, USA). Dosages were based on Silva *et al*. (2014) and Dong *et al*. (2014). For analysis of Tb, data were averaged between 1230 h and 1330 h (*n *= 5). This time frame was chosen as Tb had stabilized after increasing from handling and injection, but was 7 h before lights out when activity and Tb increase due to diurnal rhythms. Food intake (g) was split into light (11:00–16:00) and dark phases (16:00–04:00) and summed across mice (*n *= 6). To determine whether high and low S1P and SEW2871 had an effect on Tb and food intake, we used a linear mixed‐effects modelling approach using the nlme package (Pinheiro *et al*., 2016). Injection treatment (saline control, S1P high, S1P low, SEW2871 high and SEW2871 low) and activity level were used as fixed effects and mouse ID was used as a random effect. Model selection was performed by minimizing the Akaike information criterion (AIC) and model validation performed using standard residual plots (Bozdogan, 1987).

All statistical analyses were performed using the R statistical environment (R Core Team, 2015).

## Conflicts of interests

The authors declare no conflicts of interests.

## Authors' contributions

JRS conceptualized and designed the original graded CR experiment, raised the funding to execute it and was the HO project licence holder. CG performed the liver metabolite extraction (Award no. 1438803). SEM performed all prior experimental procedures related to the study. SEM and CG performed additional S1P experiment. AD, CG and DL performed the statistical analysis. CG, AD, DL, SEM and JRS interpreted the results. CG wrote the manuscript and AD, DP, DL, SEM and JRS revised it. All authors contributed to the analysis during discussions at joint meetings funded by BBSRC grant (China partnering award BB/JO20028/1).

## Data accessibility

Work towards having all the data from this series of papers online is currently ongoing. All significant metabolites in relation to CR manipulation are listed in supplementary materials Table S1. Data on the nonsignificant metabolites are freely available for anyone who requests it from the corresponding author at j.speakman@abdn.ac.uk.

## Supporting information


**Table S1** Significantly differentiated metabolites based on Benjamini‐Hochberg adjusted P‐Value ≤ 0.05. 10, 20, 30 and 40CR compared to 12AL control group. Colour indicates pathway metabolite is primarily involved in.
**Table S2** Differentiated metabolites up and downregulated for each CR group relative to the 12AL control (p ≤ 0.05).
**Table S3** Correlations between expression levels of key metabolites and circulating hormones and body weight measured after 3 months of CR.
**Table S4** Correlations between expression levels of key metabolites and markers of oxidative stress, food anticipatory activity and core body temperature.
**Table S5** Correlation of L‐carnitine and carnitine derivatives found in the liver with dROMs (Diacron reactive oxygen metabolites).
**Table S6** Model Selection for body temperature.
**Table S7** Model selection for food intake.
**Table S8** Anova table comparing models of body temperature (°C) and food intake in the dark and light cycles with and without treatment (S1P or SEW2871 injection at 100 and 200ng) as an explanatory variable.
**Table S9** Summary of linear mixed effects models of body temperature (°C) and food intake in light and dark cycles of male C57BL/6 mice injected with S1P and SEW2871.
**Data S10** Study Design.
**Data S11** S1P Experiment
