# Supplementary material for: The effects of graded levels of calorie restriction: IX. Global metabolomic screen reveals modulation of carnitines, sphingolipids and bile acids in the liver of C57BL/6 mice
Source: Aging Cell. 2017 Jan 31;16(3):529–40. doi: 10.1111/acel.12570 (PMC5418186; doi:10.1111/acel.12570)
Supplement: Supplementary file 1 — Table S1 Significantly differentiated metabolites based on Benjamini‐Hochberg adjusted P‐Value ≤ 0.05. 10, 20, 30 and 40CR compared to 12AL control group. Colour indicates pathway metabolite is primarily involved in. Table S2 Differentiated metabolites up and downregulated for each CR group relative to the 12AL control (p ≤ 0.05). Table S3 Correlations between expression levels of key metabolites and circulating hormones and body weight measured after 3 months of CR. Table S4 Correlations between expression levels of key metabolites and markers of oxidative stress, food anticipatory activity and core body temperature. Table S5 Correlation of L‐carnitine and carnitine derivatives found in the liver with dROMs (Diacron reactive oxygen metabolites). Table S6 Model Selection for body temperature. Table S7 Model selection for food intake. Table S8 Anova table comparing models of body temperature (°C) and food intake in the dark and light cycles with and without treatment (S1P or SEW2871 injection at 100 and 200ng) as an explanatory variable. Table S9 Summary of linear mixed effects models of body temperature (°C) and food intake in light and dark cycles of male C57BL/6 mice injected with S1P and SEW2871. Data S10 Study Design. Data S11 S1P Experiment [file ACEL-16-529-s001.docx]

Supplementary Materials

S1: Significantly differentiated metabolites based on Benjamini-Hochberg adjusted P-Value ≤ 0.05. 10, 20, 30 and 40CR compared to 12AL control group. Colour indicates pathway metabolite is primarily involved in.

| Metabolite | Pathway | 10CR | 20CR | 30CR | 40CR |
| --- | --- | --- | --- | --- | --- |
| L.Arabinonate | Carbohydrate Metabolism | sig | sig | sig | sig |
| O.Propanoylcarnitine | Lipids: Fatty Acyls |  | sig | sig | sig |
| Lys.Met.Phe.Trp | Peptide |  | sig | sig | sig |
| 4.Imidazolone.5.propanoate | Amino Acid Metabolism |  | sig | sig | sig |
| X.R..2.Hydroxyglutarate | Amino Acid Metabolism |  | sig | sig | sig |
| X.SP..16.0...N..hexadecanoyl..sphing.4.enine.1.phosphocholine | Lipids: Sphingolipids |  | sig | sig | sig |
| Glucosyloxyanthraquinone | Small Molecule Metabolism |  | sig | sig | sig |
| unknown.41 |  |  | sig | sig | sig |
| Phe.Val | Peptide |  | sig | sig | sig |
| unknown.52 |  |  | sig | sig | sig |
| L.Pipecolate | Amino Acid Metabolism |  | sig | sig | sig |
| X.1.Ribosylimidazole..4.acetate | Amino Acid Metabolism |  | sig | sig | sig |
| 5.Hydroxypentanoate | Lipids: Fatty Acyls |  | sig | sig | sig |
| X.PC..20.0...1.eicosanoyl.sn.glycero.3.phosphocholine | Lipids: Glycerophospholipids |  | sig | sig | sig |
| SM.d18.1.23.0. | Lipids: Sphingolipids |  | sig | sig | sig |
| X.SP..Sphing.4.enine.1.phosphate | Lipids: Sphingolipids |  | sig | sig | sig |
| 1.Hydroxy.2.aminoethylphosphonate | Amino Acid Metabolism |  | sig | sig | sig |
| 4.Nitrophenol.alpha.D.galactopyranoside | Small Molecule Metabolism |  | sig | sig | sig |
| DCI (dichloroisoproterenol) | β-adrenergic receptor antagonist |  | sig | sig | sig |
| 5.Hydroxypentanoate | Lipids: Fatty Acyls |  | sig | sig | sig |
| unknown.193 |  |  | sig | sig | sig |
| X.FA.hydroxy.9.1...4.hydroxy.2.nonenal | Lipids: Fatty Acyls |  | sig | sig | sig |
| Ethanolamine.phosphate | Amino Acid Metabolism |  | sig | sig | sig |
| 5.6.Dihydrouridine | Nucleotide Metabolism |  | sig | sig | sig |
| Capryloylglycine | Lipid Metabolism |  | sig | sig | sig |
| unknown.224 |  |  | sig | sig | sig |
| unknown.257 |  |  | sig | sig | sig |
| X.FA.methyl.oxo.5.0.2.0...2.methylene.4.oxo.pentanedioic.acid | Xenobiotics Biodegradation and Metabolism |  | sig | sig | sig |
| 5.6.Dihydrothymine | Nucleotide Metabolism |  | sig | sig |  |
| unknown.122 |  |  | sig | sig |  |
| unknown.155 |  |  | sig | sig |  |
| unknown.178 |  |  | sig | sig |  |
| Glycerophosphoglycerol | Carbohydrate Metabolism |  | sig |  | sig |
| unknown.203 |  |  | sig |  | sig |
| X.SP..22.0...N..docosanoyl..sphing.4.enine | Lipids: Sphingolipids |  | sig |  |  |
| X.PC..18.1.20.4...1..1Z.octadecenyl..2..5Z.8Z.11Z.14Z.eicosatetraenoyl..sn.glycero.3.phosphocholine | Lipids: Glycerophospholipids |  | sig |  |  |
| unknown.145 |  |  | sig |  |  |
| Betaine | Amino Acid Metabolism |  |  | sig | sig |
| O.Acetylcarnitine | Fatty Acid Transport |  |  | sig | sig |
| Succinate | Carbohydrate Metabolism |  |  | sig | sig |
| Elaidiccarnitine | Lipids: Fatty Acyls |  |  | sig | sig |
| N.Acetylglutamine | Amino Acid Metabolism |  |  | sig | sig |
| L.Threonine | Amino Acid Metabolism |  |  | sig | sig |
| X.FA..6.0...O.hexanoyl.R.carnitine | Lipids: Fatty Acyls |  |  | sig | sig |
| D.Galactosamine | Carbohydrate Metabolism |  |  | sig | sig |
| L.Methionine | Amino Acid Metabolism |  |  | sig | sig |
| X.FA..O.Palmitoyl.R.carnitine | Lipids: Fatty Acyls |  |  | sig | sig |
| unknown.39 |  |  |  | sig | sig |
| Cholesterolsulfate | Lipids: Sterol lipids |  |  | sig | sig |
| unknown.44 |  |  |  | sig | sig |
| X.GL..17.2.20.3...1..9Z.12Z.heptadecadienoyl..2..8Z.11Z.14Z.eicosatrienoyl..sn.glycerol | Lipids: Glycerolipids |  |  | sig | sig |
| Hexanoylglycine | Amino Acid Metabolism |  |  | sig | sig |
| N..octanoyl..L.homoserine | Amino Acid Metabolism |  |  | sig | sig |
| unknown.54 |  |  |  | sig | sig |
| cis.5.Tetradecenoylcarnitine | Lipids: Fatty Acyls |  |  | sig | sig |
| D.Glucose.6.sulfate | Carbohydrate Metabolism |  |  | sig | sig |
| Pro.Ser.Ser | Peptide |  |  | sig | sig |
| 2.Dehydro.3.deoxy.L.rhamnonate | Carbohydrate Metabolism |  |  | sig | sig |
| X.FA.hydroxy.10.0...N..3S.hydroxydecanoyl..L.serine | Amino Acid Metabolism |  |  | sig | sig |
| 3.tert.Butyl.5.methylcatechol | Small Molecule Metabolism |  |  | sig | sig |
| 4.Trimethylammoniobutanoate | Amino Acid Metabolism |  |  | sig | sig |
| X.ST.Hydroxy.ox..7alpha.Hydroxy.3.oxochol.4.en.24.oic.Acid | Lipids: Sterol lipids |  |  | sig | sig |
| N2.Succinyl.L.ornithine | Amino Acid Metabolism |  |  | sig | sig |
| unknown.105 |  |  |  | sig | sig |
| unknown.112 |  |  |  | sig | sig |
| unknown.114 |  |  |  | sig | sig |
| Pantetheine | Metabolism of Cofactors and Vitamins |  |  | sig | sig |
| Phosphoribosyl.AMP | Amino Acid Metabolism |  |  | sig | sig |
| unknown.147 |  |  |  | sig | sig |
| X.ST.trihydroxy.2.0...11beta.17.21.trihydroxypregn.4.ene.3.20.dione | Lipids: Sterol lipids |  |  | sig | sig |
| unknown.169 |  |  |  | sig | sig |
| Cys.Phe.Gly.His | Peptide |  |  | sig | sig |
| Metalaxyl | Fungicide |  |  | sig | sig |
| 3.Isopropylbut.3.enoic.acid | Biosynthesis of Secondary Metabolites |  |  | sig | sig |
| unknown.265 |  |  |  | sig | sig |
| unknown.267 |  |  |  | sig | sig |
| X.10S..Juvenile.hormone.III.diol | Biosynthesis of Secondary Metabolites |  |  | sig | sig |
| X.FA.hydroxy.oxo.7.0.2.0...4.hydroxy.2.oxo.Heptanedioic.acid | Amino Acid Metabolism |  |  | sig | sig |
| 3.mercapto.1.2.propanediol | Small Molecule Metabolism |  |  | sig | sig |
| unknown.322 |  |  |  | sig | sig |
| unknown.348 |  |  |  | sig | sig |
| unknown.371 |  |  |  | sig | sig |
| sn.glycero.3.Phosphocholine | Lipid Metabolism |  |  | sig |  |
| S.Acetyldihydrolipoamide | Amino Acid Metabolism |  |  | sig |  |
| Phthalate | Xenobiotics Biodegradation and Metabolism |  |  | sig |  |
| myo.Inositol.1 | Carbohydrate Metabolism |  |  | sig |  |
| Monomethyl.sulfate | Small Molecule Metabolism |  |  | sig |  |
| unknown.93 |  |  |  | sig |  |
| unknown.104 |  |  |  | sig |  |
| Xanthosine | Nucleotide Metabolism |  |  | sig |  |
| Gynocardin | Small Molecule Metabolism |  |  | sig |  |
| X.FA.methyl.14.0.2.0...3.methyl.tetradecanedioic.acid | Lipids: Fatty Acyls |  |  | sig |  |
| Calystegin.B2 | Small Molecule Metabolism |  |  | sig |  |
| 4.Sulfobenzoate | Xenobiotics Biodegradation and Metabolism |  |  | sig |  |
| unknown.207 |  |  |  | sig |  |
| Oleandolide | Biosynthesis of Polyketides and Nonribosomal Peptides |  |  | sig |  |
| unknown.215 |  |  |  | sig |  |
| unknown.229 |  |  |  | sig |  |
| unknown.258 |  |  |  | sig |  |
| Scoparone | Secondary Metabolite Phenylpropanoid |  |  | sig |  |
| N.hexanoyl.D.L.homoserine.lactone | Small Molecule Metabolism |  |  | sig |  |
| Phenylpyruvate | Amino Acid Metabolism |  |  | sig |  |
| unknown.335 |  |  |  | sig |  |
| unknown.356 |  |  |  | sig |  |
| L.Homocysteine | Amino Acid Metabolism |  |  | sig |  |
| Choline.phosphate | Lipid Metabolism |  |  |  | sig |
| Taurocholate | Lipid Metabolism |  |  |  | sig |
| L.Leucine | Amino Acid Metabolism |  |  |  | sig |
| X.FA.hydroxy.18.1...9.10.dihydroxy.12Z.octadecenoic.acid | Lipids: Fatty Acyls |  |  |  | sig |
| unknown.4 |  |  |  |  | sig |
| Ascorbate | Carbohydrate Metabolism |  |  |  | sig |
| Leu.Val | Peptide |  |  |  | sig |
| Leu.Ala | Peptide |  |  |  | sig |
| unknown.17 |  |  |  |  | sig |
| X.ST.hydrox..N..3alpha.hydroxy.5beta.cholan.24.oyl..glycine.3.sulfate | Lipids: Sterol lipids |  |  |  | sig |
| N2..D.1.Carboxyethyl..L.lysine | Amino Acid Metabolism |  |  |  | sig |
| beta.Alanyl.L.arginine | Amino Acid Metabolism |  |  |  | sig |
| Pimelylcarnitine | Lipids: Fatty Acyls |  |  |  | sig |
| N.Acetyl.L.histidine | Amino Acid Metabolism |  |  |  | sig |
| unknown.27 |  |  |  |  | sig |
| unknown.33 |  |  |  |  | sig |
| Ala.Asp.Gln | Peptide |  |  |  | sig |
| unknown.40 |  |  |  |  | sig |
| Arg.Asn.Ser.Arg | Peptide |  |  |  | sig |
| unknown.50 |  |  |  |  | sig |
| Methylimidazoleacetic.acid | Amino Acid Metabolism |  |  |  | sig |
| N.Acetyl.L.aspartate | Amino Acid Metabolism |  |  |  | sig |
| 5.Acetamidopentanoate | Amino Acid Metabolism |  |  |  | sig |
| unknown.70 |  |  |  |  | sig |
| N6.Acetyl.N6.hydroxy.L.lysine | Amino Acid Metabolism |  |  |  | sig |
| D.Glutamate | Amino Acid Metabolism |  |  |  | sig |
| 15.Keto.prostaglandinE2 | Lipids: Fatty Acyls |  |  |  | sig |
| unknown.89 |  |  |  |  | sig |
| unknown.92 |  |  |  |  | sig |
| unknown.95 |  |  |  |  | sig |
| 3..4.Hydroxyphenyl.lactate | Amino Acid Metabolism |  |  |  | sig |
| N.Acetyl.L.citrulline | Amino Acid Metabolism |  |  |  | sig |
| unknown.118 |  |  |  |  | sig |
| unknown.119 |  |  |  |  | sig |
| Aspartame | Peptide |  |  |  | sig |
| 1.2.dioctanoyl.1.amino.2.3.propanediol | Diacylglycerol Kinase Inhibitor |  |  |  | sig |
| L.Rhamnose | Carbohydrate Metabolism |  |  |  | sig |
| 2.Hydroxy.6.oxo..2..aminophenyl..hexa.2.4.dienoate.1 | Citrate/pyruvate cycle |  |  |  | sig |
| Acetylenedicarboxylate | Carbohydrate Metabolism |  |  |  | sig |
| UDP.glucuronate | Carbohydrate Metabolism |  |  |  | sig |
| Methyl.2.alpha.L.fucopyranosyl.beta.D.galactoside | Carbohydrate Metabolism |  |  |  | sig |
| unknown.130 |  |  |  |  | sig |
| N1.Methyl.2.pyridone.5.carboxamide | Metabolism of Cofactors and Vitamins |  |  |  | sig |
| N.Glycoloyl.neuraminate | Carbohydrate Metabolism |  |  |  | sig |
| unknown.141 |  |  |  |  | sig |
| unknown.146 |  |  |  |  | sig |
| unknown.148 |  |  |  |  | sig |
| 11.cis.Dehydroretinal | Lipids: Prenols |  |  |  | sig |
| unknown.161 |  |  |  |  | sig |
| p.Cresolglucuronide | Amino Acid Metabolism |  |  |  | sig |
| 4..4.Deoxy.alpha.D.gluc.4.enuronosyl..D.galacturonate | Carbohydrate Metabolism |  |  |  | sig |
| N.Acetyl.D.fucosamine | Carbohydrate Metabolism |  |  |  | sig |
| X.PG..18.1...1..9E.octadecenoyl..sn.glycero.3.phospho..1..sn.glycerol. | Lipids: Glycerophospholipids |  |  |  | sig |
| unknown.176 |  |  |  |  | sig |
| D.Ribose | Carbohydrate Metabolism |  |  |  | sig |
| Choline.1 | Amino Acid Metabolism |  |  |  | sig |
| Glu.Phe.Cys.Cys | Peptide |  |  |  | sig |
| p.aminobenzoyl.glutamate | Amino Acid Metabolism |  |  |  | sig |
| Methoprene | Xenobiotics Biodegradation and Metabolism |  |  |  | sig |
| unknown.194 |  |  |  |  | sig |
| unknown.198 |  |  |  |  | sig |
| Phenol.sulfate | Small Molecule Metabolism |  |  |  | sig |
| unknown.204 |  |  |  |  | sig |
| unknown.210 |  |  |  |  | sig |
| S.53482 | Xenobiotics Biodegradation and Metabolism |  |  |  | sig |
| Homoarginine | Amino Acid Metabolism |  |  |  | sig |
| L.Cystathionine | Amino Acid Metabolism |  |  |  | sig |
| 10.16.Dihydroxyhexadecanoic.acid | Lipids: Fatty Acyls |  |  |  | sig |
| Ala.Phe.Trp.His | Peptide |  |  |  | sig |
| unknown.246 |  |  |  |  | sig |
| unknown.251 |  |  |  |  | sig |
| Indole.3.ethanol | Amino Acid Metabolism |  |  |  | sig |
| unknown.259 |  |  |  |  | sig |
| unknown.268 |  |  |  |  | sig |
| unknown.273 |  |  |  |  | sig |
| unknown.303 |  |  |  |  | sig |
| unknown.305 |  |  |  |  | sig |
| unknown.317 |  |  |  |  | sig |
| Tocainide | Xenobiotics Biodegradation and Metabolism |  |  |  | sig |
| Stipitatate | Small Molecule Metabolism |  |  |  | sig |
| unknown.334 |  |  |  |  | sig |
| Arg.Cys.Cys.Arg | Peptide |  |  |  | sig |
| unknown.358 |  |  |  |  | sig |
| unknown.367 |  |  |  |  | sig |
| unknown.369 |  |  |  |  | sig |
| X.Z..But.2.ene.1.2.3.tricarboxylate | Carbohydrate Metabolism |  |  |  | sig |
| Caribine | Small Molecule Metabolism |  |  |  | sig |
| unknown.375 |  |  |  |  | sig |

S2: Differentiated metabolites up and downregulated for each CR group relative to the 12AL control (p ≤ 0.05).

| Upregulated | Log_2_ fold change | Downregulated | Log_2_ fold change |
| --- | --- | --- | --- |
| **10 CR** |  |  |  |
| ATP | 1.099 | urocanic acid | -1.881 |
| 5,6-dihydrothymine | 0.851 | anthranilic acid | -1.484 |
| sphingosine-1-phosphate | 0.815 | glycogen | -1.42 |
| sphingomyelin | 0.7 | phenylpyruvic acid | -0.827 |
| L-homocysteine | 0.696 | 2-aminomuconate 6-semialdehyde | -0.754 |
| S-pipecolic acid | 0.686 | maleic acid | -0.749 |
| NADH | 0.45 | 4-imidazolone-5-propanoic acid | -0.629 |
| phosphatidylethanolamine | 0.439 | D-alpha-hydroxyglutarate | -0.597 |
| L-glutamine | 0.31 | L-cystathionine | -0.418 |
| phosphatidylinositol | 0.283 | L-serine | -0.361 |
| **20CR** |  |  |  |
| S-pipecolic acid | 1.511 | urocanic acid | -1.789 |
| hydrocortisone | 1.496 | 4-imidazolone-5-propanoic acid | -1.621 |
| acetyl-coenzyme A | 1.382 | anthranilic acid | -1.503 |
| L-palmitoylcarnitine | 1.351 | ceramide | -1.107 |
| propionylcarnitine | 1.332 | 1-acylglycerophosphocholine | -1.071 |
| sphingosine-1-phosphate | 1.233 | D-alpha-hydroxyglutarate | -1.028 |
| acetyl-L-carnitine | 1.179 | ribose | -0.978 |
| D-galactosamine | 1.015 | N-caproylglycine | -0.9 |
| 5,6-dihydrothymine | 1 | phosphatidylcholine | -0.446 |
| betaine | 0.971 | octanoic acid | -0.127 |
| **30CR** |  |  |  |
| S-pipecolic acid | 2.725 | pantetheine | -2.009 |
| hydrocortisone | 2.05 | 4-imidazolone-5-propanoic acid | -1.957 |
| sphingosine-1-phosphate | 1.876 | urocanic acid | -1.674 |
| acetyl-L-carnitine | 1.637 | 1-acylglycerophosphocholine | -1.56 |
| ATP | 1.515 | anthranilic acid | -1.459 |
| taurocholic acid | 1.463 | heptanoic acid | -1.422 |
| L-palmitoylcarnitine | 1.447 | N-caproylglycine | -1.224 |
| acetyl-coenzyme A | 1.427 | D-alpha-hydroxyglutarate | -1.174 |
| propionylcarnitine | 1.392 | chenodeoxycholic acid | -1.166 |
| succinic acid | 1.355 | 2-oleoylglycerol | -1.141 |
| **40CR** |  |  |  |
| S-pipecolic acid | 3.003 | ascorbic acid | -3.459 |
| taurocholic acid | 2.301 | 4-imidazolone-5-propanoic acid | -2.554 |
| sphingosine-1-phosphate | 2.158 | phosphorylcholine | -1.582 |
| hydrocortisone | 1.806 | 1-acylglycerophosphocholine | -1.57 |
| acetyl-L-carnitine | 1.608 | N-caproylglycine | -1.564 |
| L-palmitoylcarnitine | 1.579 | pantetheine | -1.516 |
| taurochenodeoxycholate | 1.44 | L-cystathionine | -1.28 |
| D-galactosamine | 1.393 | ribose | -1.247 |
| propionylcarnitine | 1.38 | N-acetyl-L-aspartic acid | -1.217 |
| betaine | 1.333 | urocanic acid | -1.051 |

S3: Correlations between expression levels of key metabolites and circulating hormones and body weight measured after 3 months of CR. TNF-α = Tumour necrosis factor alpha, IL-6 = Interleukin 6, IGF-1 = Insulin-like growth factor 1, MUPs = Murine urinary proteins. P-values were BH adjusted and significant correlations are in bold.

|  | Leptin | | Insulin | | TNF-α | | | | IL6 | | | |  |  |
| --- | --- | --- | --- | --- | --- | --- | --- | --- | --- | --- | --- | --- | --- | --- |
|  | r | p-value | r | p-value | | | r | p-value | | | r | p-value | | |
| Ceramide | **0.597** | **0.002** | 0.413 | 0.119 | | | 0.369 | 0.202 | | | -0.303 | 0.607 | | |
|  |  |  |  |  |  |  |  |  |  |  |  |  |  |  |
| Sphingomyelin | **-0.401** | **0.048** | -0.349 | 0.163 | | | -0.108 | 0.639 | | | 0.146 | 0.937 | | |
|  |  |  |  |  |  |  |  |  |  |  |  |  |  |  |
| Sphingosine-1-phosphate | **-0.431** | **0.043** | -0.262 | 0.246 | | | -0.334 | 0.202 | | | 0.010 | 0.956 | | |
|  |  |  |  |  |  |  |  |  |  |  |  |  |  |  |
| L-carnitine | -0.327 | 0.111 | -0.282 | 0.246 | | | 0.042 | 0.814 | | | 0.105 | 0.937 | | |
|  | Resistin | | IGF-1 | | | MUPs | | | | Body Weight (g) | | | |  |
|  | r | p-value | r | p-value | | | r | p-value | | | r | p-value | | |
| Ceramide | -0.089 | 0.844 | **0.419** | **0.027** | | | **0.527** | **0.004** | | | **0.550** | **0.003** | | |
|  |  |  |  |  |  |  |  |  |  |  |  |  |  |  |
| Sphingomyelin | -0.088 | 0.844 | **-0.595** | **0.001** | | | **-0.557** | **0.003** | | | **-0.453** | **0.019** | | |
|  |  |  |  |  |  |  |  |  |  |  |  |  |  |  |
| Sphingosine-1-phosphate | 0.036 | 0.844 | **-0.597** | **0.001** | | | **-0.646** | **<0.001** | | | **-0.761** | **<0.001** | | |
|  |  |  |  |  |  |  |  |  |  |  |  |  |  |  |
| L-carnitine | -0.081 | 0.844 | **-0.429** | **0.027** | | | **-0.510** | **0.004** | | | -0.234 | 0.265 | | |
|  |  |  |  |  |  |  |  |  |  |  |  |  |  |  |

S4: Correlations between expression levels of key metabolites and markers of oxidative stress, food anticipatory activity and core body temperature. SOD = superoxide dismutase, GPX = glutathione peroxidase, OXY Test = OXY Absorbent test (antioxidant power), dROMS = Diacron reactive oxygen metabolites (measure oxidative stress), FAA = Food anticipatory activity, Tb = Body temperature. Benjamini Hochberg adjusted P-values. Significant correlations in **bold**.

|  | | Catalase | | | | SOD | | | | GPX | | | | Protein Carbonyls | | | |
| --- | --- | --- | --- | --- | --- | --- | --- | --- | --- | --- | --- | --- | --- | --- | --- | --- | --- |
|  | | r | | p-value | | r | | p-value | | r | | p-value | | r | | p-value | |
| Ceramide | | **0.454** | | **0.015** | | **0.571** | | **0.001** | | 0.240 | | 0.534 | | -0.107 | | 0.836 | |
|  |  |  |  |  |  |  |  |  |  |  |  |  |  |  |  |  |  |
| Sphingomyelin | | -0.139 | | 0.472 | | -0.268 | | 0.244 | | 0.045 | | 0.791 | | 0.131 | | 0.836 | |
|  |  |  |  |  |  |  |  |  |  |  |  |  |  |  |  |  |  |
| Sphingosine-1-phosphate | | **-0.483** | | **0.015** | | -0.270 | | 0.244 | | -0.198 | | 0.534 | | 0.344 | | 0.121 | |
|  |  |  |  |  |  |  |  |  |  |  |  |  |  |  |  |  |  |
| L-carnitine | | -0.022 | | 0.895 | | -0.212 | | 0.303 | | 0.203 | | 0.534 | | -0.061 | | 0.836 | |
|  |  |  |  |  |  |  |  |  |  |  |  |  |  |  |  |  |  |
| L-methionine | | 0.299 | | 0.159 | | 0.025 | | 0.882 | | 0.171 | | 0.534 | | -0.390 | | 0.109 | |
|  |  |  |  |  |  |  |  |  |  |  |  |  |  |  |  |  |  |
|  | OXY Test | | | | dROMS | | | | FAA | | | | Tb (°C) | | | |  |
|  | r | | p-value | | r | | p-value | | r | | p-value | | r | | p-value | |  |
| Ceramide | 0.216 | | 0.338 | | **-0.459** | | **0.013** | | **-0.412** | | **0.015** | | **0.358** | | **0.037** | |  |
| Sphingomyelin | -0.338 | | 0.167 | | **0.538** | | **0.003** | | **0.365** | | **0.024** | | -0.267 | | 0.107 | |  |
| Sphingosine-1-phosphate | -0.323 | | 0.167 | | 0.267 | | 0.183 | | **0.771** | | **<0.001** | | **-0.770** | | **<0.001** | |  |
| L-carnitine | -0.237 | | 0.338 | | **0.392** | | **0.035** | | **0.267** | | **0.089** | | -0.243 | | 0.501 | |  |
| L-methionine | -0.176 | | 0.406 | | -0.085 | | 0.715 | | **-0.400** | | **0.015** | | **0.406** | | **0.020** | |  |

S5: Correlation of L-carnitine and carnitine derivatives found in the liver with dROMs (Diacron reactive oxygen metabolites). Benjamini Hochberg adjusted p-values. Significant correlations in **bold**.

|  | dROMS | |
| --- | --- | --- |
|  | r | p-value |
| L-carnitine | **0.392** | **0.021** |
| Cis-5-Tetradecenoylcarnitine | 0.299 | 0.080 |
| O-Propanoylcarnitine | **0.458** | **0.013** |
| [FA] O-Palmitoyl-R-carnitine | **0.492** | **0.012** |
| O-Butanoylcarnitine | 0.230 | 0.165 |
| Elaidic Carnitine | **0.392** | **0.021** |
| Trans-2-dodecenoylcarnitine | **0.415** | **0.021** |

S6: **Model Selection for body temperature.** Model 6 was chosen as the best model based on the AIC score. Model 6 included Temperature as its response variable, activity as the explanatory variable and mouse ID as random effect.

| Model | DF | AIC | BIC | Log Likelihood | Test | Log Ratio | P-value |
| --- | --- | --- | --- | --- | --- | --- | --- |
| 1 | 21 | 34.93279 | 83.60004 | 3.533605 |  |  |  |
| 2 | 7 | 14.34631 | 30.56873 | -0.173155 | 1 vs 2 | 7.41352 | 0.9176 |
| 3 | 12 | -12.7252 | 15.08469 | 18.36258 | 2 vs 3 | 37.07147 | <.0001 |
| 4 | 8 | -18.4957 | 0.04423 | 17.24784 | 3 vs 4 | 2.22949 | 0.6936 |
| 5 | 3 | 11.65122 | 18.60369 | -2.825612 | 4 vs 5 | 40.1469 | <.0001 |
| 6 | 4 | -23.4954 | -14.2255 | 15.74771 | 5 vs 6 | 37.14664 | <.0001 |

**Model 1:** Response variable = temperature, explanatory variable = treatment, random effects = treatment and ID

**Model 2:** Response variable = temperature, explanatory variable = treatment, random effect = ID

**Model 3:** Response variable = temperature, explanatory variables = treatment, activity and their interaction, random effect = ID

**Model 4:** Response variable = temperature, explanatory variables = treatment and activity, random effect = ID

**Model 5:** Response variable = temperature, explanatory variable = none, random effect = ID

**Model 6:** Response variable = temperature, explanatory variable = activity, random effect = ID

S7: **Model selection for food intake.** For the light phase the best model was model 4 based on the AIC score. Model 4 included the sum of food intake between 11:00 – 16:00 as its response variable, a single fixed effect, the intercept, and mouse ID as random effect. For the dark phase, the best model was Model 5 based on the AIC score. The response variable was the sum of food intake between 16:00 – 04:00 with a single fixed effect, the intercept, and mouse ID as random effect.

| Light |  |  |  |  |  |  |
| --- | --- | --- | --- | --- | --- | --- |
| Model | DF | AIC | BIC | Log Likelihood | Log Ratio | P-value |
| 1 | 7 | -145.674 | -130.453 | 79.837 |  |  |
| 2 | 12 | -144.794 | -118.701 | 84.397 | 9.120 | 0.104 |
| 3 | 8 | -145.413 | -128.018 | 80.706 | 7.381 | 0.117 |
| 4 | 3 | -153.277 | -146.754 | 79.639 | 2.136 | 0.830 |
| 5 | 4 | -152.961 | -144.263 | 80.480 | 1.684 | 0.194 |
| Dark |  |  |  |  |  |  |
| Model | DF | AIC | BIC | Log Likelihood | Log Ratio | P-value |
| 1 | 21 | 52.02915 | 97.69128 | -5.014573 |  |  |
| 2 | 7 | 27.79324 | 43.01395 | -6.896621 | 3.764095 | 0.9967 |
| 3 | 12 | 31.37975 | 57.4724 | -3.689875 | 6.413492 | 0.268 |
| 4 | 8 | 29.71039 | 47.10549 | -6.855196 | 6.330642 | 0.1758 |
| 5 | 3 | 23.1384 | 29.66156 | -8.569201 | 3.428011 | 0.6343 |
| 6 | 4 | 25.13779 | 33.83534 | -8.568894 | 0.000616 | 0.9802 |

**Light**

**Model 1:** Response variable = food intake, explanatory variable = treatment, random effect = ID

**Model 2:** Response variable = food intake, explanatory variables = treatment, activity and their interaction, random effect = ID

**Model 3:** Response variable = food intake, explanatory variables = treatment and activity, random effect = ID

**Model 4:** Response variable = food intake, explanatory variable = none, random effect = ID

**Model 5:** Response variable = food intake, explanatory variable = activity, random effect = ID

**Dark**

**Model 1:** Response variable = food intake, explanatory variable = treatment, random effects = treatment and ID

**Model 2:** Response variable = food intake, explanatory variable = treatment, random effect = ID

**Model 3:** Response variable = food intake, explanatory variables = treatment, activity and their interaction, random effect = ID

**Model 4:** Response variable = food intake, explanatory variables = treatment and activity, random effect = ID

**Model 5:** Response variable = food intake, explanatory variable = none, random effect = ID

**Model 6:** Response variable = food intake, explanatory variable = activity, random effect = ID

S8: Anova table comparing models of body temperature (°C) and food intake in the dark and light cycles with and without treatment (S1P or SEW2871 injection at 100 and 200ng) as an explanatory variable. In the body temperature model activity is also used as an explanatory variable. In all models mouse ID was used as the random effect.

|  | Explanatory Variable | DF | AIC | BIC | Log Likelihood | Likelihood Ratio | P-value | |
| --- | --- | --- | --- | --- | --- | --- | --- | --- |
| Body Temperature | Treatment + Activity | 8 | -18.496 | 0.0442 | 17.248 |  |  |  |
|  | Activity | 4 | -23.495 | -14.226 | 15.747 | 3 | 0.558 |  |
| Food Intake Light | Treatment | 7 | -145.674 | -130.453 | 79.837 |  |  |  |
|  | ~ 1 | 3 | -153.277 | -146.754 | 79.639 | 0.397 | 0.983 |  |
| Food Intake Dark | Treatment | 7 | 27.793 | 43.014 | -6.897 |  |  |  |
|  | ~ 1 | 3 | 23.138 | 29.662 | -8.569 | 3.345 | 0.502 |  |

S9: Summary of linear mixed effects models of body temperature (°C) and food intake in light and dark cycles of male C57BL/6 mice injected with S1P and SEW2871. In the body temperature model activity was used as a fixed effect. Mouse ID was used as the random effect for all models.

| Fixed Effects |  |  |  |  |  |  | Random Effects | |
| --- | --- | --- | --- | --- | --- | --- | --- | --- |
|  |  | F | DF | P | β | SE | Standard Deviation | |
| Body Temperature | Intercept | 271393.1 | 69 | <0.001 | 35.33551 | 0.072891 | Intercept | 0.144858 |
|  | Activity | 50.48 | 69 | <0.001 | 0.05009 | 0.00705 | Residual | 0.18407 |
| Food Intake Light | Intercept | 7.917 | 60 | 0.007 | 0.047 | 0.017 | Intercept | 0.032 |
|  |  |  |  |  |  |  | Residual | 0.068 |
| Food Intake Dark | Intercept | 5025.358 | 60 | <0.001 | 2.446 | 0.035 | Intercept | 0 |
|  |  |  |  |  |  |  | Residual | 0.276 |

S10: **Study Design**

Mice were purchased from Charles River (Ormiston, UK). Free access to water was provided. Body mass and food intake were recorded daily, immediately prior to feeding. Over a two week baseline period a number of measures were taken including dual X-ray absorptiometry (DXA) for body composition, glucose tolerance tests (GTT) and resting metabolic rate. Mice were randomly allocated into 6 experimental groups matched for body mass. Prior to culling all baseline measures were repeated. After three months of CR mice were culled approximately 4 hours prior to lights out between 14:00 to 18:00 by a terminal CO_2_ overdose. Blood sample was collected by heart puncture. The liver was divided into seven pieces which were individually snap frozen in liquid nitrogen in cryovials to avoid freeze/thaw artefacts. Tissues were stored at -80°C. Any apparent disease states were recorded.

**S11: S1P Experiment**

Male C57BL/6 mice were obtained from Charles River UK at 12 weeks of age (n=6). Mice were maintained on a 12:12h light-dark cycle (lights off at 16:00h with 20 minutes dawn/dusk period). Mice were individually housed in a temperature controlled room (21±1°C) with AL access to water and high carbohydrate open source diet (D12450B: Research diets, NJ, USA). Body mass and food intake were recorded daily.

MiniMitters (OR, USA) were implanted intraperitoneally a week after arrival followed by a three week recovery period. These use the VitalView^TM^ telemetry and data acquisition system to measure core Tb and activity every minute described previously (Gamo et al., 2013). Mice were moved into BioDAQ (Research Diets Inc, New Brunswick, USA) cages 10 days after surgery and given 12 days to acclimatise to eating out of hoppers before experimental procedures began. The BioDAQ system was used to monitor the animals feeding behaviour recording amount eaten, bout length and time of feeding.

S1P was dissolved in NaOH at 1mg/ml of NaOH, and then made up into a 1µg/ml with saline. SEW4871 was dissolved in ethanol at 1mg/ml, and then made up to 1µg/ml with saline. For injections 100µl was used for 100ng injections and 200µl was injected for 200ng.

**
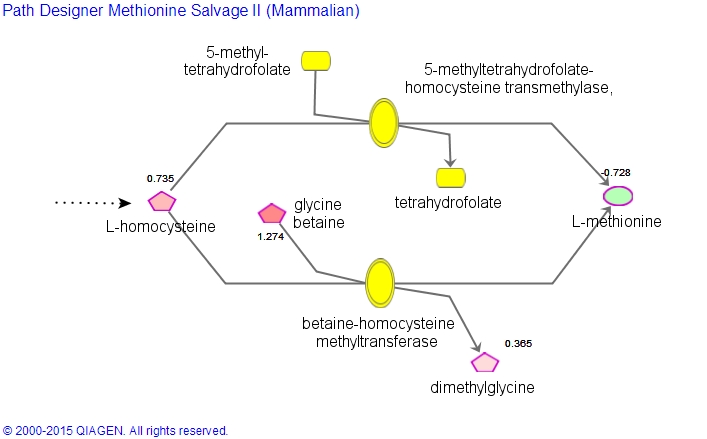

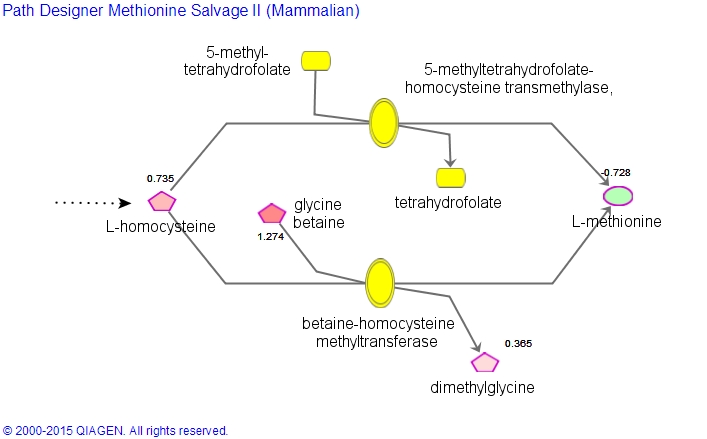
**
